# Cigarette consumption estimates for 71 countries from 1970 to 2015: systematic collection of comparable data to facilitate quasi-experimental evaluations of national and global tobacco control interventions

**DOI:** 10.1136/bmj.l2231

**Published:** 2019-06-19

**Authors:** Steven J Hoffman, Jessica Mammone, Susan Rogers Van Katwyk, Lathika Sritharan, Maxwell Tran, Safa Al-Khateeb, Andrej Grjibovski, Elliot Gunn, Sara Kamali-Anaraki, Ben Li, Mathura Mahendren, Yasmeen Mansoor, Navneet Natt, Ejike Nwokoro, Harkanwal Randhawa, Melodie Yunju Song, Kelsey Vercammen, Carolyne Wang, Julia Woo, Mathieu JP Poirier

**Affiliations:** 1 Global Strategy Lab, Dahdaleh Institute for Global Health Research, Faculty of Health and Osgoode Hall Law School, York University, 4700 Keele Street, Dahdaleh Building 2120, Toronto, Ontario, M3J 1P3 Canada; 2 School of Epidemiology and Public Health, Faculty of Medicine, University of Ottawa, Ottawa, Canada; 3 Department of Global Health & Population, Harvard T H Chan School of Public Health, Harvard University, Boston, MA, USA; 4 Department of Health Research Methods, Evidence, and Impact, Faculty of Health Sciences, McMaster University, Hamilton, Canada; 5 International School of Public Health, Northern State Medical University, Arkhangelsk, Russia; 6 Department of Health Policy and Management, Al Farabi Kazakh National University, Almaty, Kazakhstan; 7 Department of Economics, Faculty of Social Sciences, McMaster University, Hamilton, Canada; 8 Norwegian Institute of Public Health, Oslo, Norway; 9 School of Kinesiology and Health Science, Faculty of Health, York University, Toronto, Canada

## Abstract

**Objectives:**

To collect, appraise, select, and report the best available national estimates of cigarette consumption since 1970.

**Design:**

Systematic collection of comparable data.

**Setting and population:**

71 of 214 countries for which searches for national cigarette consumption data were conducted, representing over 95% of global cigarette consumption and 85% of the world’s population.

**Main outcome measures:**

Validated cigarette consumption data covering 1970-2015 were identified for 71 countries. Data quality appraisal was conducted by two research team members in duplicate, with greatest weight given to official government sources. All data were standardised into units of cigarettes consumed per year in each country, a detailed accounting of data quality and sourcing was prepared, and all collected data and metadata were made freely available in an open access dataset.

**Results:**

Cigarette consumption fell in most countries over the past three decades but trends in country specific consumption were highly variable. For example, China consumed 2.5 million metric tonnes (MMT) of cigarettes in 2013, more than Russia (0.36 MMT), the United States (0.28 MMT), Indonesia (0.28 MMT), Japan (0.20 MMT), and the next 35 highest consuming countries combined. The US and Japan achieved reductions of more than 0.1 MMT from a decade earlier, whereas Russian consumption plateaued, and Chinese and Indonesian consumption increased by 0.75 MMT and 0.1 MMT, respectively. These data generally concord with modelled country level data from the Institute for Health Metrics and Evaluation and have the additional advantage of not smoothing year-over-year discontinuities that are necessary for robust quasi-experimental impact evaluations.

**Conclusions:**

Before this study, publicly available data on cigarette consumption have been limited; they have been inappropriate for quasi-experimental impact evaluations (modelled data), held privately by companies (proprietary data), or widely dispersed across many national statistical agencies and research organisations (disaggregated data). This new dataset confirms that cigarette consumption has decreased in most countries over the past three decades, but that secular country specific consumption trends are highly variable. The findings underscore the need for more robust processes in data reporting, ideally built into international legal instruments or other mandated processes. To monitor the impact of the WHO Framework Convention on Tobacco Control and other tobacco control interventions, data on national tobacco production, trade, and sales should be routinely collected and openly reported.

## Introduction

Tobacco consumption is one of the world’s leading preventable causes of mortality, accounting for six million preventable deaths each year.^1^ Although the global prevalence of daily smoking has decreased in both men and women from 1980 to 2012,^2^ the absolute number of smokers has increased from 720 million people in 1980 to almost one billion in 2012.^3^ Moreover, pressure from the tobacco industry has further encouraged an increase in cigarette smoking.^3^ ^4^ The World Health Organization predicts that the cumulative number of tobacco related deaths will increase to one billion in the 21st century (up from 100 million in the 20th century) unless global tobacco control measures are implemented rapidly.^5^

The WHO Framework Convention on Tobacco Control (FCTC) was adopted in May 2003 with the goal of reducing harmful tobacco consumption, preventing smoking among children, and counteracting the influence that tobacco companies have long maintained through advertising, promotion, and sponsorships. However, 15 years after the FCTC was signed by nearly every country, there remains a lack of publicly available data suitable for conducting rigorous impact evaluations, hindering research in this area. The limited existing data that are publicly available on cigarette consumption have been inappropriate for quasi-experimental impact evaluations (modelled data), held privately by companies (proprietary data), or widely dispersed across many national statistical agencies and research organisations (disaggregated data).^2^

Different methods of quantifying cigarette consumption have advantages and limitations. Measuring self reported cigarette consumption through surveys such as the Global Youth Tobacco Survey and national health surveys offers a detailed cross sectional data source that is an invaluable part of the tobacco control ecosystem. Nevertheless, these survey data can be under-reported, and under-reporting can vary depending on cultural and gender norms in different countries.^6^ ^7^ Furthermore, data coverage is limited to the years in which nationally representative surveys are conducted, and survey questions can vary from country to country or even from survey to survey. Many of these challenges can be overcome by use of administrative cigarette sales data systematically collected by many countries in the world. Although some discrepancies between sales and actual consumption can emerge because of stockpiling, spoilage, and illicit trade, sales data are considered to be the most accurate, internationally comparable measure of cigarette consumption.^7^ ^8^

The Institute for Health Metrics and Evaluation (IHME) has previously published a dataset of smoking prevalence and consumption worldwide and by country, from 1980 to 2012.^2^ However, the IHME data cannot be used for rigorous quasi-experiments, which test descriptive causal hypotheses about manipulable interventions in order to draw counterfactual inferences about what would have happened in the absence of intervention, but without random assignment.^9^ Much of the IHME data were gathered from proprietary sources, after which gaps in the data were filled by imputation, estimates synthesised with a two stage linear model, and selected with a Gaussian process regression using each country’s gross domestic product and regional dummy variables.^2^ ^10^ This data-generating process creates smoothed data series, which cannot be used for quasi-experiments that leverage breaks or discontinuities in data.

As a result, the extent of the tobacco epidemic in each country is not always known and the impact of an intervention on tobacco consumption cannot be rigorously evaluated, preventing policy makers from responding appropriately when prioritising limited health budgets. Despite being the most prominent example of a tobacco control intervention, the FCTC has not yet been empirically evaluated at the global level using a quasi-experimental design. Several studies have examined the impact of this treaty on the adoption of domestic tobacco control policies^11^ and on smoking prevalence in individual countries or regions,^12^ ^13^ ^14^ ^15^ ^16^ ^17^ ^18^ ^19^ ^20^ ^21^ but no studies have explored the relation between the FCTC and global tobacco consumption, owing to a lack of comparable data. Indeed, we cannot even confirm whether the many evidence based policies on tobacco control pursued as a result of this treaty have translated into real-world impact for the many countries where such policies have been adopted but not necessarily fully implemented or not directly evaluated.^22^ ^23^ ^24^ ^25^ ^26^ ^27^ ^28^ ^29^ ^30^ ^31^ These vital questions regarding the effectiveness of national and global interventions have largely remained unanswered due to a lack of comparable data.

This study outlines a systematic and reproducible effort undertaken to collect, appraise, select, and report comparable best-available national estimates of cigarette consumption, as estimated from sales and implied sales, covering 1970-2015 for as many countries as possible. These data build on earlier work conducted by WHO and the American Cancer Society, and are drawn from cigarette production, trade, and sales statistics, as well as direct consumption estimates, all of which are better suited for making global comparisons than modelled data.^7^ ^32^ The results of this effort are now available in an open access dataset covering 71 countries that account for over 95% of the world’s cigarette consumption and 85% of the world’s population (https://dataverse.scholarsportal.info/dataverse/iccd). This dataset is unique in the analyses and comparisons of cigarette consumption trends that it enables and should facilitate the quasi-experimental evaluation of national and global tobacco control interventions, including the FCTC and the policies it promotes.

## Methods

### Data collection

Systematic searches were conducted to collect cigarette consumption data for all countries from 1970 to 2015. Fourteen research assistants participated in data collection for 214 countries between May 2014 and May 2016. Specifically, an initial adaptive effort was undertaken to gather data on the production, trade, and sales of cigarettes from each country’s national statistical agency (box 1). The decision to focus on cigarettes (as opposed to electronic cigarettes, water tobacco, chewing tobacco, loose leaf tobacco, or alternative tobacco products such as beedis and kreteks) was made for pragmatic reasons to ensure direct comparability of the data among countries and over time. We searched freely available publications, such as statistical yearbooks, for data using key terms including (but not limited to) “cigarette,” “production,” “external trade,” “manufacturing”, “industry,” and “tobacco.” If complete data were not found on the national statistical website, we used an internet search engine (Google) to locate specific government ministries that might contain relevant information (eg, ministries of finance, economy, revenue, industry, manufacturing, trade, customs). We also searched academic databases to identify research publications related to cigarette consumption, which were used to trace the source information or to contact the authors to request their data. Data from international and non-governmental organisations (such as the United Nations, Euromonitor, and GlobalData) were used if national government data could not be found. These secondary data aggregators are usually less transparent about their data sources, but often obtain figures from national statistical agencies, other non-governmental organisations, or even directly from tobacco companies.

Box 1 **Data collection and quality appraisal processes in systematic search**

**Data collection process**

Data sourced following the steps below:

- National statistical agency
- National government ministries (eg, finance, trade)
- Publications on tobacco consumption
- Data from intergovernmental organisations (eg, United Nations)
- Data from non-governmental organisations (eg, Euromonitor, GlobalData)
- Email national agency with telephone follow-up
- Contact experts for missing data.

**Data quality appraisal process**

Data appraised following the steps below:

- Compile production, trade, and sales data
- Calculate implied sales (production plus imports minus exports)
- Determine intersource consistency
- Consider contextual factors of the country or year
- Contact experts to clarify discrepancies
- Select most reliable data source for each year
- Document reasons for selecting each data point
- Assess for gaps and inconsistencies
- Assign data confidence level for each country.

When possible, searches were conducted in each country’s main language by multilingual members of the research team or by volunteer translators who were recruited to assist with the search process. For some jurisdictions (eg, Cuba, Iran, Taiwan), volunteer translators searched physical documents and books for data. If a native speaker was unavailable, Google Translate was used to search internet resources. We used a standardised email script when contacting authors of past studies and experts listed on the websites of national statistical agencies and government departments to request access to their data (appendix A). Often, the same email was sent to multiple experts in different departments to ensure retrieval of all available information. In the rare case of a fee request in exchange for data, a request to waive the fee was made or the principal investigators decided on a case-by-case basis whether payment was feasible and appropriate. If a reply was not received after one week, a follow-up email was sent to the same contact requesting the data again. If no reply was received one week after the follow-up email, a third email was sent indicating that the team would be happy to speak with them by telephone instead. A telephone call requesting data was then made at a time that corresponded with business hours of the contact country (standardised telephone script included in appendix A). If an email response was not received from a non-English speaking country, volunteer translators were recruited to contact the agencies by telephone. The team was limited by the infeasibility of recruiting translators for all languages, and therefore, was unable to contact certain non-English-speaking countries by telephone. Translators were identified for Albanian, Arabic, Bengali, French, Russian, and Spanish languages, but not for Hungarian, Khmer, Lao, Latvian, Malay, Portuguese, or Ukrainian languages (appendix B lists the countries that required volunteer translators to contact by telephone).

When units of measurement differed within and between countries, we standardised the data to be equivalent to one metric tonne, or one million cigarette sticks. For instance, some countries reported production data in kilograms or in cigarette units (sticks), in which case an equivalent weight of one gram per cigarette was used because it is the most common choice and allows for more conservative and internationally comparable estimates.^33^ As another example, Chile reported production as a manufacturing index with several different base years. Although the number of years of data available varied across countries (table 1), we did not impute any missing data, allowing future data users to decide whether to model any gaps in data according to their research questions and methods. We also did not present data aggregated at the region level, because this also required modelling or imputation to account for significant missing data in some countries and regions. Finally, data from former East and West Germany were combined for the duration of the study period, and one year was chosen as a break point at which to divide countries of the former Soviet Union (1996), Yugoslavia (1990), and Czechoslovakia (1992).

**Table 1:** Data collected for top 20 cigarette-consuming countries for 2010

Rank	Country	Country information			Cigarette consumption			No of years of data collected			
		FCTC date*	Population aged ≥15		Per capita	Gross		Production	Imports	Exports	Consumption
1	China	Ratified: 11 Oct 2005	1 107 440 301		2145	2 375 260		27	46	45	45
2	Russian Federation	Accessioned: 3 June 2008	121 840 686		3214	391 635		19	19	19	19
3	United States	Signed: 10 May 2004	248 664 927		1235	307 205		46	46	45	45
4	Japan	Accepted: 8 June 2004	110 399 140		1904	210 200		44	45	45	45
5	Indonesia	Not a participant	171 822 771		1130	194 203		23	43	45	43
6	Philippines	Ratified: 6 June 2005	61 768 596		1877	115 947		25	44	45	45
7	India	Ratified: 5 February 2004	850 680 556		117	99 619		24	45	44	46
8	Brazil	Ratified: 3 November 2005	148 202 902		654	96 918		25	46	28	46
9	Turkey	Ratified: 31 December 2004	52 875 455		1800	95 195		44	44	31	31
10	Ukraine	Ratified: 6 June 2006	39 308 685		2410	94 727		18	19	19	19
11	Republic of Korea	Ratified: 16 May 2005	41 117 785		2243	92 225		25	45	45	45
12	Vietnam	Ratified: 17 December 2004	67 438 304		1344	90 628		24	40	24	21
13	Italy	Ratified: 2 July 2008	51 233 059		1698	87 000		45	39	45	45
14	Germany	Ratified: 16 December 2004	69 559 301		1201	83 565		46	44	45	45
15	Spain	Ratified: 11 January 2005	39 798 668		1820	72 431		45	43	45	45
16	Egypt	Ratified: 25 February 2005	55 838 165		1277	71 277		24	45	45	42
17	Pakistan	Ratified: 3 November 2004	108 526 264		599	64 985		24	27	39	45
18	Poland	Ratified: 15 September 2006	32 803 848		1747	57 320		45	45	35	25
19	France	Approved: 19 October 2004	51 341 275		1067	54 797		45	39	45	45
20	United Kingdom	Ratified: 16 December 2004	51 618 652		876	45 235		46	44	45	45

* Date of ratification or signing of the WHO Framework Convention on Tobacco Control (FCTC).

### Data appraisal

Data collected for each country were appraised by at least two research team members in duplicate for intersource consistency, with more weight being given to official government sources (box 1). Whenever possible, we compared sales data with implied sales data calculated from domestic production plus imports minus exports of cigarettes, as a secondary measure.^8^ If conflicting data arose, we contacted country experts to seek their advice on which source to prioritise. In some instances, this process elucidated issues with certain sources, such as the exclusion of small to medium manufacturers, inclusion of alternative tobacco products, or double counting imports or exports. Some discontinuities in data could be due to changes in volume of smuggling, stockpiling of imports, or falsifying exports.^34^ ^35^ A detailed country-by-country accounting of data quality appraisal and selection for each year is presented in appendix C, and the data sources chosen for each year are identified in the open access dataset. Data confidence was again evaluated by two team members and determined to be high if data covering nearly the entire study period were found and were corroborated with alternative sources. Data confidence was considered moderate if a temporal gap in data emerged or if no corroborating data could be found, and considered low if both of these issues emerged. This process was conducted for every country in order of gross cigarette consumption (from highest to lowest as per earlier estimates from IHME^2^) until enough data were collected to cover 95% of worldwide consumption. Although there is no reliable measure of illicit international tobacco trade, we have identified countries known to be sources, conduits, and destinations for illicit tobacco in appendix C,^36^ although tobacco companies have repeatedly overstated the magnitude of illicit tobacco markets.^37^

### Data analysis

Descriptive statistics were conducted to present an overview of the data contained in the dataset. The open access dataset contains data on annual cigarette sales, production, imports, and exports, organised by country and year. The unit of measurement and data source were listed for each data point. The data collected in this systematic effort for the top five cigarette-consuming countries were also compared with equivalent IHME consumption estimates to assess their level of concordance.^2^

### Patient and public involvement

Patients were not involved in this study. Students were heavily engaged in the data collection process. The resulting open access dataset on cigarette consumption is now freely available to the public at https://dataverse.scholarsportal.info/dataverse/iccd. Data and findings resulting from follow-up studies will be actively disseminated through conference presentations, publications in academic journals, plain language policy notes, personalised briefings to leading global tobacco control organisations, and commentary in news media.

## Results

The open access dataset was broadly laid out in four sections: sales, production, imports, and exports, with up to three different sources compiled for each country year. The most reliable estimates of both sales and estimated sales (that is, production + imports − exports) as determined by the data appraisal process were provided, followed by the most reliable consumption figure, a per capita consumption rate (in units of cigarette sticks per person per year for all individuals aged 15 and over), and the data source for every country year.

**Fig 1:**
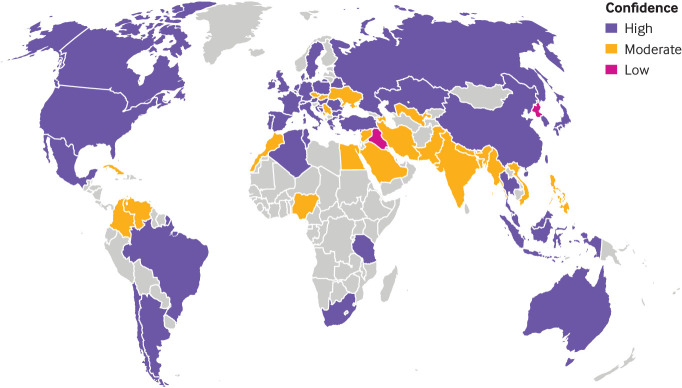
Countries (n=71) included in the study dataset, shaded according to appraised confidence in the data

Of the 214 countries for which systematic searches for national cigarette consumption data were conducted, complete or validated data were identified for 71 countries (seven in Africa, 24 in Asia, one in the Caribbean, 30 in Europe, six in Latin America, two in North America, one in Oceania; appendix D). Three were former countries (the Soviet Union, Yugoslavia, and Czechoslovakia). Additionally, Taiwan, while not formally recognised by the United Nations as a distinct country, was included as a separate jurisdiction. Data confidence was classified as high for 46 countries (four in Africa, 11 in Asia, 23 in Europe, five in Latin America, two in North America, and one in Oceania), moderate for 23 (three in Africa, 11 in Asia, one in the Caribbean, seven in Europe, and one in Latin America), and low for two (both in Asia; fig 1).

**Fig 2:**
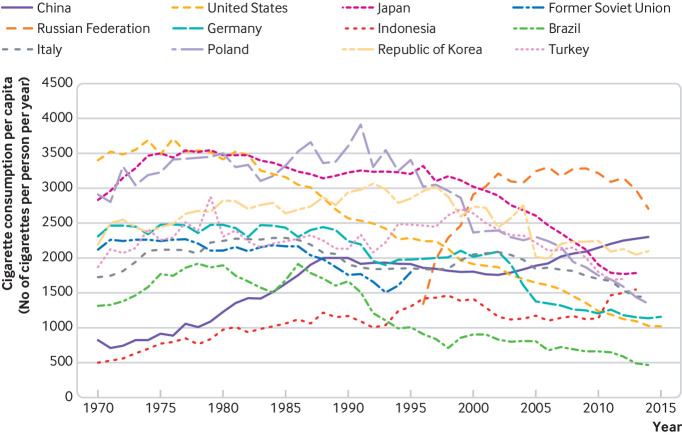
Trends in cigarette consumption per capita for the top 10 cigarette-consuming countries, from 1970 to 2015

As summarised in figure 2, a steady and general decline in cigarette consumption per capita was observed, from around 1985 in five of the top 10 cigarette-consuming countries: US, Japan, Poland, Brazil, and Germany. By contrast, consumption per capita rose steadily in China and Indonesia. Mixed progress of increases and decreases in cigarette consumption, or a plateauing of progress, was observed in the remaining top 10 countries (Russia, South Korea, and Italy).

**Fig 3:**
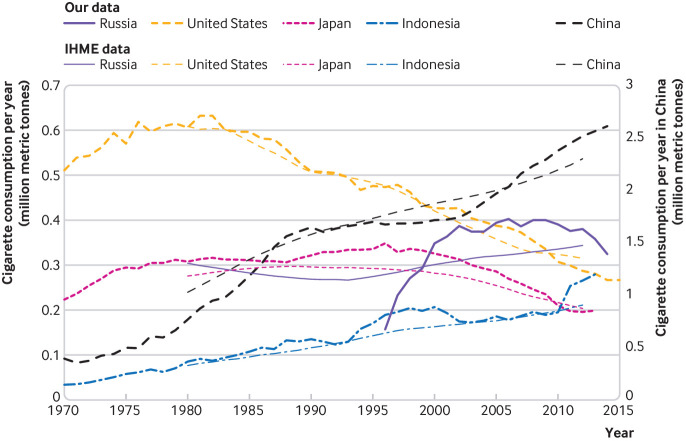
Comparison of Institute for Health Metrics and Evaluation (IHME) estimates of cigarette consumption, for the top five cigarette-consuming countries from 1970 to 2015, with data compiled through the present study’s systematic collection of data. Chinese consumption data are presented on the right hand axis to enhance visibility of trends for other countries

China was the world’s leading consumer of cigarettes, with over 2.5 million metric tonnes (MMT) consumed in 2013—more than the next 40 highest consuming countries combined. The results for the US and Japan represented reductions of more than 0.1 MMT from a decade earlier, whereas Russian consumption plateaued, and Chinese and Indonesian consumption increased by 0.75 MMT and 0.1 MMT, respectively. The comparison of the top five cigarette-consuming countries with corresponding IHME consumption estimates^2^ showed a general concordance on levels and trends, but with a clear difference in granularity and smoothness of data (fig 3). Although the IHME’s linear approximation tracked the US and Indonesia’s actual consumption quite closely, IHME’s linear models for China, Japan, and especially Russia were not accurate representations of these countries’ non-linear trends in cigarette consumption. Therefore, someone using IHME data for Russia would mistakenly see a steady and moderate increase in consumption from 1993 to 2012 after moderate decreases from 1980 to 1993. By contrast, verified yearly data showed that Russian consumption more than doubled between 1996 and 2002 before plateauing and eventually decreasing after 2010. This discrepancy is not simply a difference in level, but is a qualitatively distinct result with potentially different policy implications.

## Discussion

### Principal findings

This systematic collection of data showed that internationally comparable data on cigarette consumption are dispersed and that data quality varies across countries. Some of the challenges included unavailable data; incomplete data; data reported in a manner incompatible with the research aims; unreachable contacts at national agencies; and, at times, language barriers. Comparisons with modelled cigarette consumption estimates indicated that the present study’s data better captures and is more representative of actual year-over-year changes in cigarette consumption, which would not be expected to follow linear trends in any given year (even if a linear trend might be expected over many years). However, modelled results might still be helpful for estimating consumption in countries without any data and for research requiring data from all countries. In any case, this study’s new open access dataset of systematically collected, appraised, and selected comparable national estimates of cigarette consumption is an important step towards conducting more rigorous impact evaluations of national and global tobacco control interventions.

General trends in cigarette consumption per capita vary widely by country and region. African countries’ consumption varied in both level and trend, with north African countries consuming more than sub-Saharan countries. The US, Canada, and Australia all showed similar continuous declines in consumption since the early 1980s, while Latin American and Caribbean countries had more modest declines. Western and northern European countries had a nearly continuous reduction in consumption since 1970, but southern and eastern European countries showed widely varying patterns of consumption, with some countries’ consumption increasing substantially (eg, Russia and Belarus). Central and western Asian countries’ consumption levels were among the highest in the world, and in many cases increased rapidly over the past decade. By contrast with other east Asian countries, China’s consumption increased after a short lived plateau just before 2000. Although south Asian countries’ consumption levels were much lower than those of east Asian countries, these levels have not decreased over time. Finally, southeast Asian consumption trends varied, with some increases in several of the region’s most populous countries (eg, Indonesia and Vietnam).

### Policy implications

The results of this study underscore the need for more robust data reporting processes. Many countries that currently have minimal reporting of tobacco production or tax receipts of tobacco sales should implement robust data collection and reporting processes, as New Zealand’s 1990 Smoke-free Environments Act has done.^38^ ^39^ To monitor the impact of the FCTC and other tobacco control interventions on cigarette consumption, country level data on tobacco production, trade, and sales should be routinely collected and openly reported. The FCTC contains no standardised requirements for data reporting beyond periodic reports on the implementation of the treaty, despite the great importance of these outcome data for both the health of states parties’ populations and to monitoring the FCTC itself. Instead, there should be an international legal obligation among FCTC states parties to systematically and transparently collect annual data on tobacco production, trade, and sales, disaggregated by type of tobacco, and openly reported by countries and international institutions. Mandated biennial reports submitted by FCTC states parties to this treaty’s secretariat are often of poor quality and lack standardisation across countries; these reports could be strengthened to become a more robust data gathering mechanism.^40^ Such data would provide researchers and the public with the tools to track the impact of their governments’ policies and to progress towards protecting present and future generations from the poor health, social, environmental and economic consequences of tobacco consumption and exposure to tobacco smoke.

### Strengths and limitations

The primary strength of this study was its systematic data collection effort with transparent methods. We used an adaptive search strategy that focused on national statistical websites and government ministries and involved contacting country and subject matter experts to locate missing data. Past studies have suggested that official data from national sources are more reliable than estimates derived from undisclosed algorithms or imputation.^1^ ^2^ Our search had no language restriction, because we recruited multilingual members of the research team and volunteer translators, allowing us to collect data from many sources. Another strength of the study was that we estimated cigarette consumption data via aggregate production, trade and sales data. While population based surveillance of tobacco use is important for understanding tobacco use behaviours and establishing prevalence levels, past studies have shown that survey respondents often report consuming less tobacco than they actually do.^6^ ^7^ Ideally, globally comparable yearly estimates of tobacco use prevalence and smoking intensity would be available for every country worldwide, but in the absence of such data, sales and implied sales are the most reliable measures available for many research applications.^7^ ^8^ All retrieved data are presented in an open access dataset, which will enable researchers to conduct studies using verified and comparable cigarette consumption data.

For limitations, we sometimes had difficulties in obtaining data owing to unreachable country experts, invalid telephone numbers and email addresses, or national statistical agencies not collecting the relevant data. In these cases, other avenues for securing the data were explored, including through academic institutions within each country or third party research publications with relevant data. We were unable to verify data for many countries with low cigarette consumption due to the time needed to conduct the systematic collection and verification process. Research involving these countries’ cigarette consumption should use modelled data, such as estimates compiled by IHME,^2^ although limits to what can be done with these data using quasi-experimental methods should be considered. We were also unable to consistently quantify illicit international flows of cigarettes or consumption by non-citizens, meaning our estimates represented only official counts of legal cigarette purchases. To ensure direct comparability among countries and over time, data collection was limited to cigarette consumption and did not include electronic cigarettes, water tobacco, chewing tobacco, loose leaf tobacco, or alternative tobacco products such as beedis and kreteks, which are commonly used tobacco products in some countries. Finally, the reliability of each country’s consumption estimates depends on the accuracy and precision with which each country collects production, trade, and sales data. Anyone using this new dataset should carefully examine the metadata (including the source and reliability of each country’s data) before conducting analyses, to ensure that the data were measured with sufficient precision for the intended purpose.

### Future research directions

Better quality data on cigarette consumption are an important first step in evaluating national and global tobacco control interventions, and will contribute to current and future efforts to combat the global tobacco epidemic. Few verified consumption data were collected for African countries because of poor data quality and comparatively low levels of aggregate consumption, but anticipated population growth, improvements in living standards, and tobacco industry targeting mean that this data gap must be addressed. Future research could include analysing global trends in cigarette consumption across regions, income level, and other stratifying factors; evaluating the impact of various tobacco control interventions on cigarette consumption with robust quasi-experimental designs; calculating the impact of tobacco control interventions on smoking related deaths; and, if combined with economic data, assessing the cost effectiveness of these interventions.

A companion study has already used the open access dataset to complete a quasi-experimental impact evaluation of the FCTC using interrupted time series analysis and in-sample forecast event modelling,^41^ which represents the first time, to our knowledge, that these methods have been used to evaluate an international law.^42^ This impact evaluation—and future evaluations using this dataset—can further tobacco control efforts by not only answering key questions about the effectiveness of the FCTC (thus providing further momentum for its implementation or the pursuit of alternative strategies) but also generating crucial evidence that can help shape targeted tobacco control interventions more broadly.

**What is already known on this topic**

In 2014, the Institute for Health Metrics and Evaluation (IHME) published modelled country level data on cigarette consumption covering 187 countries from 1980 to 2012; to our knowledge, it is the only open access dataset of verified international cigarette consumption
Proprietary data sources are known to exist (eg, Euromonitor, GlobalData, and internal documents at various international and non-governmental organisations), but are generally not available to researchers or the public

**What this study adds**

An open access dataset of internationally comparable estimates of cigarette consumption was developed covering 71 countries from 1970 to 2015, accounting for over 95% of the world’s cigarette consumption and 85% of the world’s population
By comparison with IHME’s modelled estimates, the current dataset better captures and is more representative of actual year-over-year changes in cigarette consumption, which would not be expected to follow linear trends in any given year
Use of this dataset allows for quasi-experimental evaluations of national and global interventions on tobacco control, including the WHO Framework Convention on Tobacco Control and the important policies it promotes

## Web extra

Extra material supplied by authors

**Web appendix A: **Standardised data request scripts

**Web appendix B:** List of countries that required volunteer translators to contact by telephone

**Web appendix C:** Country-by-country descriptions of data appraisal and selection for all years, by gross consumption

**Web appendix D:** Data collected and verified across countries and years

**Web appendix E:** Glossary of all acronyms used in the database

## Notes

Contributors: SJH conceived the study, led its design as principal investigator, and, with MJPP, supervised implementation. SJH, JM, SRVK, LS, MT, and MP led the analysis and preparation of this manuscript. AG, EG, BL, MM, YM, NN, EN, HR, KV, CW, SA-K, MYS, and JW assisted with data collection, supervised by SJH, JM, LS, MT, and MJPP. All authors reviewed, edited, and approved the final manuscript. SJH and MJPP are the guarantors. The lead author (SJH) and corresponding author (MJPP) attest that all listed authors meet authorship criteria and that no others meeting the criteria have been omitted.

Ethical approval: This study was approved by McMaster University’s Hamilton Integrated Research Ethics Board (14-378). We attest that we have obtained appropriate permissions and paid any required fees for use of copyright protected materials.

Data sharing: The full dataset can be accessed at: https://dataverse.scholarsportal.info/dataverse/iccd. Additional data and methodological details can be found in the appendices. All inquiries regarding the dataset can be made to the corresponding author.

The lead author (SJH) affirms that the manuscript is an honest, accurate, and transparent account of the study being reported; that no important aspects of the study have been omitted; and that any discrepancies from the study as originally planned have been explained.

Publisher’s note: Published maps are provided without any warranty of any kind, either express or implied. BMJ remains neutral with regard to jurisdictional claims in published maps.

## References

[1] World Health Organization. WHO global report on trends in prevalence of tobacco smoking. Geneva: World Health Organization; 2015. https://apps.who.int/iris/bitstream/10665/156262/1/9789241564922_eng.pdf

[2] Ng M Freeman MK Fleming TD Smoking prevalence and cigarette consumption in 187 countries, 1980-2012. JAMA 2014;311:183-92. 10.1001/jama.2013.284692 24399557

[3] Beaglehole R Bonita R Yach D Mackay J Reddy KS A tobacco-free world: a call to action to phase out the sale of tobacco products by 2040. Lancet 2015;385:1011-8. 10.1016/S0140-6736(15)60133-7 25784348

[4] United States Department of Health and Human Services. The health consequences of smoking - 50 years of progress: a report of the Surgeon General. Atlanta: US Department of Health and Human Services, Centers for Disease Control and Prevention, National Centre for Chronic Disease Prevention and Health Promotion; 2014. https://www.ncbi.nlm.nih.gov/books/NBK179276/pdf/Bookshelf_NBK179276.pdf

[5] World Health Organization. WHO report on the global tobacco epidemic, 2013. Geneva: World Health Organization; 2013. https://apps.who.int/iris/bitstream/10665/85381/1/WHO_NMH_PND_13.2_eng.pdf

[6] Hatziandreu EJ Pierce JP Fiore MC Grise V Novotny TE Davis RM The reliability of self-reported cigarette consumption in the United States. Am J Public Health 1989;79:1020-3. 10.2105/AJPH.79.8.1020 2751017 PMC1349899

[7] Guindon GE, Boisclair D. Past, current and future trends in tobacco use. Washington, DC: World Bank; 2003 [cited 2018 Aug 29] p1-62. Report No. 29265. http://documents.worldbank.org/curated/en/374771468128405516/pdf/292650Guindon1Past10current10whole.pdf

[8] International Agency for Research on Cancer. Methods for evaluating tobacco control policies. Lyon, France: International Agency for Research on Cancer; 2008 [cited 2018 Dec 5]. (IARC Handbooks of Cancer Prevention; vol. 12). http://publications.iarc.fr/Book-And-Report-Series/Iarc-Handbooks-Of-Cancer-Prevention/Methods-For-Evaluating-Tobacco-Control-Policies-2008

[9] Shadish WR, Cook TD, Campbell DT. Experimental and quasi-experimental designs for generalized causal inference. Boston, MA, US: Houghton, Mifflin and Company; 2002. xxi, 623.

[10] James SL Gubbins P Murray CJ Gakidou E Developing a comprehensive time series of GDP per capita for 210 countries from 1950 to 2015. Popul Health Metr 2012;10:12. 10.1186/1478-7954-10-12 22846561 PMC3487911

[11] Hoffman SJ Tan C Overview of systematic reviews on the health-related effects of government tobacco control policies. BMC Public Health 2015;15:744. 10.1186/s12889-015-2041-6 26242915 PMC4526291

[12] Thrasher JF Reynales-Shigematsu LM Baezconde-Garbanati L Promoting the effective translation of the framework convention on tobacco control: a case study of challenges and opportunities for strategic communications in Mexico. Eval Health Prof 2008;31:145-66. 10.1177/0163278708315921 18390866 PMC6109969

[13] Lv J Su M Hong Z Implementation of the WHO Framework Convention on Tobacco Control in mainland China. Tob Control 2011;20:309-14. 10.1136/tc.2010.040352 21493635

[14] Lunze K Migliorini L Tobacco control in the Russian Federation--a policy analysis. BMC Public Health 2013;13:64. 10.1186/1471-2458-13-64 23339756 PMC3732080

[15] Tumwine J Implementation of the framework convention on tobacco control in Africa: current status of legislation. Int J Environ Res Public Health 2011;8:4312-31. 10.3390/ijerph8114312 22163209 PMC3228573

[16] Sebrié EM Schoj V Travers MJ McGaw B Glantz SA Smokefree policies in Latin America and the Caribbean: making progress. Int J Environ Res Public Health 2012;9:1954-70. 10.3390/ijerph9051954 22754484 PMC3386598

[17] Singh PK MPOWER and the Framework Convention on Tobacco Control implementation in the South-East Asia region. Indian J Cancer 2012;49:373-8. 10.4103/0019-509X.107738 23442401

[18] Katanoda K Jiang Y Park S Lim MK Qiao Y-L Inoue M Tobacco control challenges in East Asia: proposals for change in the world’s largest epidemic region. Tob Control 2014;23:359-68. 10.1136/tobaccocontrol-2012-050852 23596197 PMC4078676

[19] Martínez C Martínez-Sánchez JM Robinson G Bethke C Fernández E Protection from secondhand smoke in countries belonging to the WHO European Region: an assessment of legislation. Tob Control 2014;23:403-11. 10.1136/tobaccocontrol-2012-050715 23596198

[20] Mir H Roberts B Richardson E Chow C McKee M Analysing compliance of cigarette packaging with the FCTC and national legislation in eight former Soviet countries. Tob Control 2013;22:231-4. 10.1136/tobaccocontrol-2012-050567 23047889

[21] Usmanova G Mokdad AH Results of the Global Youth Tobacco Survey and implementation of WHO Framework Convention on Tobacco Control in former Soviet Union countries. Int J Public Health 2013;58:217-26. 10.1007/s00038-012-0433-2 23224517

[22] Bader P Boisclair D Ferrence R Effects of tobacco taxation and pricing on smoking behavior in high risk populations: a knowledge synthesis. Int J Environ Res Public Health 2011;8:4118-39. 10.3390/ijerph8114118 22163198 PMC3228562

[23] Hammond D Health warning messages on tobacco products: a review. Tob Control 2011;20:327-37. 10.1136/tc.2010.037630 21606180

[24] Brinn MP Carson KV Esterman AJ Chang AB Smith BJ Mass media interventions for preventing smoking in young people. Cochrane Database Syst Rev 2010;(11):CD001006. 21069667 10.1002/14651858.CD001006.pub2

[25] DiFranza JR Which interventions against the sale of tobacco to minors can be expected to reduce smoking? Tob Control 2012;21:436-42. 10.1136/tobaccocontrol-2011-050145 21994275

[26] Guillaumier A Bonevski B Paul C Anti-tobacco mass media and socially disadvantaged groups: a systematic and methodological review. Drug Alcohol Rev 2012;31:698-708. 10.1111/j.1465-3362.2012.00466.x 22571783

[27] Moodie C Stead M Bauld L McNeill A Angus K Hinds K Plain tobacco packaging: A systematic review. Public Health Research Consortium, 2012, http://phrc.lshtm.ac.uk/papers/PHRC_006_Final_Report.pdf.

[28] Mozaffarian D Afshin A Benowitz NL American Heart Association Council on Epidemiology and Prevention, Council on Nutrition, Physical Activity and Metabolism, Council on Clinical Cardiology, Council on Cardiovascular Disease in the Young, Council on the Kidney in Cardiovasc Population approaches to improve diet, physical activity, and smoking habits: a scientific statement from the American Heart Association. Circulation 2012;126:1514-63. 10.1161/CIR.0b013e318260a20b 22907934 PMC3881293

[29] Reda AA Kotz D Evers SMAA van Schayck CP Healthcare financing systems for increasing the use of tobacco dependence treatment. Cochrane Database Syst Rev 2012;(6):CD004305. 22696341 10.1002/14651858.CD004305.pub4

[30] Wilson LM Avila Tang E Chander G Impact of tobacco control interventions on smoking initiation, cessation, and prevalence: a systematic review. J Environ Public Health 2012;2012:961724. 10.1155/2012/961724 22719777 PMC3376479

[31] Hamilton FL Greaves F Majeed A Millett C Effectiveness of providing financial incentives to healthcare professionals for smoking cessation activities: systematic review. Tob Control 2013;22:3-8. 10.1136/tobaccocontrol-2011-050048 22123941

[32] Shafey O Dolwick S Guindon GE Tobacco control country profiles. Atlanta Am Cancer Soc, 2003: 356.

[33] Organisation for Economic Cooperation and Development. OECD health statistics 2018 definitions, sources and methods. 2018. http://www.oecd.org/els/health-systems/Table-of-Content-Metadata-OECD-Health-Statistics-2018.pdf

[34] Collin J Legresley E MacKenzie R Lawrence S Lee K Complicity in contraband: British American Tobacco and cigarette smuggling in Asia. Tob Control 2004;13(Suppl 2):ii104-11. 10.1136/tc.2004.009357 15564212 PMC1766170

[35] Gruber J, Sen A, Stabile M. Estimating price elasticities when there is smuggling: the sensitivity of smoking to price in Canada. National Bureau of Economic Research; 2002 [cited 2018 Sep 19]. Report No: 8962. https://www.nber.org/papers/w8962 10.1016/S0167-6296(03)00058-4 12946461

[36] World Health Organization. The protocol to eliminate illicit trade in tobacco products: questions and answers. Framework Convention on Tobacco Control. [cited 2019 Apr 1]. https://www.who.int/fctc/protocol/faq/en/

[37] Gallagher AWA Evans-Reeves KA Hatchard JL Gilmore AB Tobacco industry data on illicit tobacco trade: a systematic review of existing assessments. Tob Control 2019;28:334-45. 30135114 10.1136/tobaccocontrol-2018-054295 PMC6580768

[38] McNeill A Hammond D Gartner C Whither tobacco product regulation? Tob Control 2012;21:221-6. 10.1136/tobaccocontrol-2011-050258 22345253

[39] Laugesen M Swinburn B New Zealand’s tobacco control programme 1985-1998. Tob Control 2000;9:155-62. 10.1136/tc.9.2.155 10841851 PMC1748326

[40] Hoffman SJ Rizvi Z WHO’s undermining tobacco control. Lancet 2012;380:727-8. 10.1016/S0140-6736(12)61402-0 22920746

[41] Hoffman SJ Poirier MJP Rogers Van Katwyk S Baral P Sritharan L Impact of the WHO Framework Convention on Tobacco Control on global cigarette consumption: quasi-experimental evaluations using interrupted time series analysis and in-sample forecast event modelling. BMJ 2019;365:l2287. 10.1136/bmj.l2287 PMC6582266 31217191

[42] Hoffman SJ Hughsam M Randhawa H International law’s effects on health and its social determinants: protocol for a systematic review, meta-analysis, and meta-regression analysis. Syst Rev 2016;5:64. 10.1186/s13643-016-0238-0 27084338 PMC4833910

