# Supplementary material for: Cigarette consumption estimates for 71 countries from 1970 to 2015: systematic collection of comparable data to facilitate quasi-experimental evaluations of national and global tobacco control interventions
Source: BMJ. 2019 Jun 19;365:l2231. doi: 10.1136/bmj.l2231 (PMC6582269; doi:10.1136/bmj.l2231)
Supplement: Supplementary file 1 — Web appendix A: Standardised data request scripts [file hofs048711.ww1.pdf]

Appendix A. Standardized data request scripts

<u>Email script</u>

"Dear Sir/Madam,

I am a researcher at the Centre for Health Economics and Policy Analysis at McMaster University in Canada. I am currently working on a project examining national tobacco and alcohol production and consumption trends around the world. Your website was very helpful in identifying ______, however we were unable to access ________. We would greatly appreciate any data you have available on ____________. If you have any further questions or concerns, please do let me know. If it would be easier to speak over the phone, I can give you a call at a time that is most convenient for you.

I look forward to your reply."

<u>Telephone script</u>

If the call is answered:
"Hello, my name is _____ and I am a researcher at the Centre for Health Economics and Policy Analysis at McMaster University in Canada. I am calling in order to request some data regarding tobacco and alcohol production and consumption in your country. Is there a person I can speak to regarding this data request?"

Once directed to the appropriate contact:
"I am calling on behalf of a research team at the Centre for Health Economics and Policy Analysis at McMaster University in Canada. Our team is currently working on a project examining national tobacco and alcohol production and consumption trends around the world. We were wondering if you could assist us with our project by providing us with data regarding tobacco and alcohol consumption, price, sales, production, imports, and exports in your country since 1985?"

Voice message to leave if no one answers the telephone:
"Hello, my name is _____ and I am calling on behalf of a research team at the Centre for Health Economics and Policy Analysis at McMaster University in Canada. Our team is currently working on a project examining national tobacco and alcohol production and consumption trends around the world, and we were wondering if you would be able to provide us with relevant data on this topic.
Please email me at ____________ so that we can discuss further details regarding my request.
Thank you very much for your time and I look forward to hearing back from you."
