# Supplementary material for: Cigarette consumption estimates for 71 countries from 1970 to 2015: systematic collection of comparable data to facilitate quasi-experimental evaluations of national and global tobacco control interventions
Source: BMJ. 2019 Jun 19;365:l2231. doi: 10.1136/bmj.l2231 (PMC6582269; doi:10.1136/bmj.l2231)
Supplement: Supplementary file 2 — Web appendix B: List of countries that required volunteer translators to contact by telephone [file hofs048711.ww2.pdf]

Appendix B. List of countries that required volunteer translators to contact by telephone

Language	Translator Recruited?	Countries
**French**	Yes	Haiti, Senegal, Equatorial Guinea, Gabon, Mali, Niger, Togo
**Russian**	Yes	Russia, Kazakhstan, Kyrgyz
**Albanian**	Yes	Kosovo
**Spanish**	Yes	Paraguay Peru, Venezuela, Dominican, El Salvador, Guatemala, Honduras, Nicaragua
**Arabic**	Yes	Tunisia, Bahrain, Iraq, Syria
**Bengali**	Yes	Bangladesh
**Portuguese**	No	East Timor, Sao Tome & Principe
**Hungarian**	No	Hungary
**Latvian**	No	Latvia
**Ukrainian**	No	Ukraine
**Dzongkha**	No	Bhutan
**Malay**	No	Brunei
**Khmer**	No	Cambodia
**Lao**	No	Lao PDR
