# Supplementary material for: Cigarette consumption estimates for 71 countries from 1970 to 2015: systematic collection of comparable data to facilitate quasi-experimental evaluations of national and global tobacco control interventions
Source: BMJ. 2019 Jun 19;365:l2231. doi: 10.1136/bmj.l2231 (PMC6582269; doi:10.1136/bmj.l2231)
Supplement: Supplementary file 3 — Web appendix C: Country-by-country descriptions of data appraisal and selection for all years, by gross consumption [file hofs048711.ww3.pdf]

Appendix C. Country-by-country descriptions of data appraisal and selection for all years, by gross consumption. Superscript labels identify countries identified by the WHO as major sources (a), conduits (b), and destinations (c) for illicit tobacco. All acronyms are identified in the glossary [Appendix E].

Country	World Consumption Rank (2010)	Consumption (M, 2010)	Data Confidence (Reviewer 1)	Data Confidence (Reviewer 2)	Description of data quality (synthesis of two independent appraisers)
China[a]	1	2,375,260	High	Moderate	USDA production estimates and FAO import/export data are used from 1970-2004, after which official NBSC production numbers are used. FAO and UN import/export data follow the same trend, allowing the use of UN data for 2014. Although NBSC sales data are available, the large discrepancy between these figures and every other import/export source discourages their use.
Russian Federation[a]	2	391,635	High	High	Russian Federation data begins at 1996, as with all former Soviet Union countries. UN production, import, and export data are consistent with ISS sales estimates, FAO import/export data, and Euromonitor sales estimates. Early CIS production data are higher than all other estimates, but these appear to be a continuation of Soviet Union-wide production numbers. ERC sales estimates diverge somewhat in the mid-2000s, but their production numbers track UN data closely throughout.
United States[c]	3	307,205	High	High	High quality TBOT sales data was chosen over FTC data because these figures include all cigarettes sold including imported cigarettes and cigarettes produced or sold on tribal lands, rather than just those produced by large manufacturers. These sales data are supported by USDA, TTB, UN, ERC, and FAO data.
Japan	4	210,200	High	High	Official sales data from Statistics Japan are used since 1985. ISS sales data are used from 1970, supported by 23 years of exact data overlap. All secondary production data from ERC and USDA, as well as import/export data from UN, FAO, Statistics Japan, and ERC, are in strong agreement.

Indonesia[b]	5	194,203	High	High	Continuous official production data from the Ministry of Agriculture and, beginning in 2006, from the Ministry of Finance, are used in conjunction with UN import/export data, which are consistent with FAO import/export data. ERC estimates are moderately higher but follow a similar trend.
Philippines[b]	6	115,947	Moderate	Moderate	Official BIR sales data are only available from 2008-2014. USDA production data with UN and FAO import/export data are used until 1989, after which ERC is the only source for estimates. UN and ERC import/export data are identical, and FAO tracks very closely when data overlap.
India[b]	7	99,619	Moderate	Moderate	Official CSO production data are used from 1970-2010 and are identical to UN production figures. CSO production estimates obtained from the industrial index of production are used from 2011-2014. Official Department of Commerce import/export figures are used for every year available (1996-2014). FAO import/export figures are used from 1986-1995 because they more closely track official figures than UN data. UN import/export data are used from 1970-1985. ERC estimates are significantly higher for every year, although this may be due to the inclusion of "beedi" cigarettes.
Brazil[c]	8	96,918	High	High	Official production data from Receita Federal available since 2000 track ERC data closely. UN production data are implausibly low for 1999, 2000 and 2002. USDA production data are used for earlier years, and UN import/export data used throughout is mostly consistent with FAO data.
Turkey[b]	9	95,195	High	Moderate	Official sales data obtained directly from TEKEL until 2000, and from Ministry of Finance from 2004-2012. The three-year gap is filled with implied production from TurkStat industrial production index with UN import/export data. UN production data are available, but a sudden doubling of production in 2002 is not supported

					by ERC or TurkStat. UN and FAO import/export data are almost identical.
Ukraine[a]	10	94,727	Moderate	Moderate	Official UKRSTAT production data available from 2003 is almost identical to UN production data for five years of overlap. UN production data are used from 1996-2003, and UN import/export data are available for every year. UN, FAO, and WHO import/export are not identical, but follow the same overall trend. Early ISS sales data are only available for first eight years, and are rounded estimates, not official figures. ADIC figures, although not exact, support the use of UN figures.
Korea, Republic of	11	92,225	High	High	Official sales data from Statistics Korea is available from 1989 and is highly consistent with UN, ERC, and FAO production and import/export data. USDA production with UN import/export data are used for earlier years.
Viet Nam[b]	12	90,628	Moderate	Moderate	Official production data are available for every year, except for a five-year gap from 1990-1994, but ERC and ADB have nearly identical estimates for these years and ADB data for 1995-1997 are identical to official data. Although ERC estimate large quantities of illicit importation of cigarettes (backed up by FAO), UN and official data are used, resulting in low importation quantities and increasingly large export figures.
Italy[c]	13	87,000	High	High	Official sales compiled by ISS from 1970-2011 and by the EC from 2002-2014. These two sources are identical for ten years of overlap, and are supported by USDA, ERC, Euromonitor, UN, and FAO production and import/export data.
Germany[c]	14	83,565	High	High	Official sales data from Statistisches Bundesamt are available for unified Germany since 1991. These figures are exactly reproduced by ISS and the EC, and are supported by production and import/export data from USDA, ERC, Euromonitor, UN, and FAO. ISS sales data from both East and West Germany are combined for years 1970-

					1989, and the gap year of 1990 is filled using USDA production data and FAO import/export data.
Spain[c]	15	72,431	High	High	Official sales compiled by ISS from 1970-2001 and by the EC from 2002-2014. These two sources are nearly identical for nine years of overlap, and are supported by USDA, ERC, Euromonitor, UN, and FAO production and import/export data.
Egypt[b]	16	71,277	Moderate	Moderate	Early data is only available from UN (production) and FAO (import/export), but these are supported by figures aggregated by the WHO from 1970 and 1980. A five-year gap from 1990-1994 is filled with ERC data, which is again supported by a WHO 1990 estimate. UN production and import/export data is then used until 2000, after which official production data from CAPMAS statistical yearbooks are used for production figures. There is a significant divergence in production estimates in recent years, with ERC and an alternate CAPMAS data source publishing much higher estimates, but unofficial source of these estimates and clear rounding of numbers led to the decision to use official data supported by UN production data. There is also a discrepancy between FAO and UN import/export data in recent years, but there are gaps in FAO data and ERC import/export estimates support the use of UN data.
Pakistan[c]	17	64,985	Moderate	Moderate	State Bank of Pakistan production data are used since 1996. Since ERC data track these official figures exactly, they are used from 1990-1995. UN is the preferred import/export data source, and FAO is used for earlier years. Pakistan statistical yearbook, Ministry of Industries, and Euromonitor estimate similar, but not identical, production figures. UN and FAO import/export figures are the same, except for one obvious unit mistake by FAO in 1999.
Poland[b]	18	57,320	High	High	Sales compiled by ISS are used from 1970-2012, and EC sales data for 2013-2014. In this case, there is divergence between ISS and EC figures, although they do converge in

					later years. Official production data from CSO with UN import/export data align with ISS figures, so these are used. CSO figures are supported by USDA, ERC, and Euromonitor. There are some discrepancies between ERC, FAO, and UN import/export data; and even the UN has revised their estimates over the years. However, all combinations support the decision to use ISS sales data.
France[c]	19	54,797	High	High	Official sales compiled by ISS from 1970-2001 and by the EC from 2002-2014. These two sources are identical for nine years of overlap, and are supported by USDA, ERC, Euromonitor, UN, and FAO production and import/export data; although this supporting data tends to diverge from sales data in later years. However, official data published by the INPES public health agency are identical to the ISS and EC figures.
United Kingdom[c]	20	45,235	High	High	Official HM Revenue & Customs (HMRC) sales data is used from 1987-2014, and ISS compilation of HMRC sales data are used from 1970. These are supported by USDA, Euromonitor, and ERC production data, along with UN, ERC, and Euromonitor import/export data.
Argentina[b]	21	41,762	High	High	Official sales data is available from the Ministerio de Agricultura Ganadería y Pesca as one continuous series from 1970-2014. This is well supported by independent sales figures from the National Institute of Statistics and Censuses, and from USDA and ERC production with UN and FAO import/export data.
Thailand	22	39,779	High	High	Production and import/export data from the UN is used from 1970-1993, at which point official TTM/Ministry of Finance production and import/export data are available. ERC and Euromonitor production data are almost identical to the official production data. UN import data are somewhat higher than official figures, and UN export figures are significantly higher than official figures, although not large enough to drive overall consumption.

Taiwan	23	37,890	High	High	Official sales data from NTA are available until 2014 except for 2000-2001. Although data are unstable for these years, ERC estimates are almost identical to official data and are almost identical to production data, so these data are used to fill the missing years. ERC, national statistics data, and FAO import/export data all track very closely.
Mexico	24	34,985	High	High	Early data from 1970-1993 use UN production and import/export data, which are supported by four years of overlap with ERC data. From 1994-2008 production data compiled in a report titled "The Economics of Tobacco and Tobacco Taxation in Mexico" are used, because UN production data contains missing and anomalously low figures from 1994-2006. This decision is supported by very similar estimates produced by the governmental organization Centro de Estudios de las Finanzas Públicas and the ERC. Beginning in 2003 official SIAVI import/export figures are used, which are nearly identical to those produced by the UN and FAO. Finally, 2014-2015 production figures are taken from the official INEGI Encuesta Mensual de la Industria Manufacturera.
Saudi Arabia	25	33,213	Moderate	Low	There is no domestic production of cigarettes in Saudi Arabia, and yearly imports can make data more volatile than other countries. Because of this effect, even official data is extremely unstable before 2000, but improve significantly thereafter. UN import/export data are used for every year, except 1997. ERC data are identical, and FAO data are the same except for years that are imputed. 1997 has an anomalously high import volume, but the official CDS data are identical to FAO and ERC estimates.
Syrian Arab Republic[b]	26	32,757	Moderate	Low	Official CBSSYR production figures are used from 2004-2011, and UN production data is used for every year prior. Official import/export data are almost identical to UN and FAO for years of overlap (2004-2011), but data become sparser prior to 1990. ERC estimates are lower than all

					other sources. All data point to a significant increase in consumption since 2009.
Canada[c]	27	32,404	High	High	High quality sales data from Statistics Canada are available for both cigarettes and fine cut tobacco beginning in 1980. There is a significant exception from 1991-1993, where several studies have documented- a significant rise in smuggling and falsified exports following a policy change. In order to represent actual consumption more accurately, production plus imports, but not minus exports is used for these years. USDA production with UN import/export data are used from 1970-1980. These are supported by UN, ERC, and Euromonitor production, and FAO import/export data.
Iraq[c]	28	29,700	Low	Low	Although ERC estimates are available starting in 1990, data are clearly imputed, and no secondary data source could be found to support these estimates.
Iran, Islamic Republic of[c]	29	28,417	Moderate	Moderate	UN production data was used (1970-2009) because it is exactly reproduced for every year of overlap with official AMAR production data (1996, 2001, 2002-2011), although this diverges significantly from ERC production estimates. FAO import/export data are used, except for 1994-2000, where data are clearly imputed (e.g. 15000 MT for 1998-2000). Import/export data from Iran Tobacco Co. are available for 1992-2015, but import data diverge significantly from both UN and FAO estimates and result in wild swings in consumption from year to year. ERC estimates are used to fill gaps of 1994-1997 and 2000, but imputed data from 1998-1999 result in a two-year data gap.
Serbia and Montenegro[b]	30	25,745	Moderate	Moderate	Official data from a SORS production index are used from 2000-2015, which closely track sporadic production data available from UN, SORS, Euromonitor, and EuroStat. UN production data are used from the first year of 1991. FAO import/export data are used from 1991-1999, after which official data from SORS is used, with the exception of a gap in 2003 which is filled with Euromonitor figures.

Belarus[a]	31	25,428	High	High	UN production and import/export data are strongly supported by ERC estimates and data sent from the Belarussian statistical agency (2002-2013). UN data are not available for 1996, and ERC is not used because it is an imputed value.
Greece[b]	32	24,005	High	Moderate	Official sales compiled by ISS from 1970-2012 and by the EC from 2002-2014. These two sources are identical for eleven years of overlap, and are supported by ERC and UN production data and FAO import/export data.
Kazakhstan	33	23,868	High	High	Official KazStat production data are available from 2004 to 2013, and UN production is used for 1996-2003, all with UN import/export data. There is perfect agreement in five years of overlap (2004-2008), and these data are supported by ERC production data and FAO import/export data.
Bangladesh	34	23,677	Moderate	Moderate	Official BBS sales data are used from 1997-2013, with the exception of 2007-2009 which uses BBS production data with UN import/export data. UN production and import/export data are used for every year prior to 1997. This is supported by identical ERC production data, although ERC estimates a much large import volume. However, FAO figures are strongly support the UN data. Production data from Universal Leaf are at least two times higher than all other sources, which may include smaller producers not captured by other sources, but lack of support by any other source and only eleven years of data make the source a secondary estimate. Finally, the implied consumption from the 2010 Bangladesh Household Income and Expenditure Survey of approximately 26,000 M cigarettes supports the use of UN and BBS data.
Nigeria[b]	35	23,130	Moderate	Moderate	Production and import/export data from many sources are sporadic. UN production data with FAO import/export data are used from 1987-1989, after which ERC consumption estimates are used. The few years that have UN or official NBS production data as well as import/export data (1990-1995, 2001-2003) support the

					use of ERC estimates. Euromonitor production estimates are imputed and inconsistent with every other source.
Australia	36	21,970	High	High	Two sources of sales data differ in that ABS measures dutiable pre-rolled cigarettes only and ISS includes roll-your-own cigarettes, resulting in higher consumption figures. USDA and ERC production (with UN, FAO, and ERC import/export data) all support the higher ISS figures, so these are used.
South Africa[c]	37	21,606	High	High	Official SARS sales data available from 1970-2015 is supported by ERC, USDA, and Euromonitor production data, and UN and FAO import/export data.
Czech Republic[c]	38	21,060	Moderate	Moderate	ISS compilation of legal sales are used from 1997-2014, and official estimates implied by official per-capita smoking rates are used from 1993. European commission figures are slightly higher, but ISS data are further supported by ERC production with UN import/export data.
Romania[c]	39	18,604	High	High	Official INSSE production figures are used with UN import/export data from 1998-2014, and UN production figures are used from 1991-1997. ISS sales estimates are used from 1970-1990. EC and ERC sales estimates track official figures closely, although ISS figures are somewhat more stable in later years.
Algeria[b]	40	17,810	High	High	Official ONS production data along with UN import/export are used for every year available (1979-2012), and UN production is used for 2013. UN production data matches official data for all 18 years of overlap. ERC estimates are higher than all others, but these include an attempt to quantify cigarettes produced by merchants and small enterprises. FAO data supports UN import/export figures.
Morocco	41	16,730	Moderate	Moderate	USDA sales estimates are used from 1970-1994, after which ERC production estimates are used. Although no official production data could be obtained, five years of overlap between USDA and ERC data track closely. Import/export data are also uncertain, with official data

					from the Office des Changes available from 2006-2015 estimating larger import volumes than UN, FAO, and ERC.
Korea, Democratic People's Republic of	42	16,675	Low	Low	Imputed ERC estimates are available from 1990-2013, but with no secondary data, these are not included.
Uzbekistan	43	16,000	Moderate	Moderate	Production data are consistent between all sources (ERC, Euromonitor, UN), but import/export flows reported by ERC and Euromonitor diverge significantly. ERC data are used to maintain consistency.
Chile[b]	44	14,709	High	High	Official INE production is used for every year available (1990-2014), and UN production data are used for every year prior. All 20 years of overlap between these two sources, as well as ERC production data are identical. UN import/export data are used and is identical to FAO import/export data.
Portugal[c]	45	14,199	High	High	EC sales data are available from 2002-2014. UN production and import/export data are used for every year prior. Although ISS sales data are available, these are estimates compiled from a variety of sources including USDA, official statistics, and KPMG. FAO import/export data are identical to UN data for every year of overlap.
Tunisia[b]	46	13,997	High	High	Official TNS sales data are available for 1995-2007. Official TNS production data (from production index) is used from 2008-2013, and UN production data prior to 1995. ERC production data are identical to UN data, and closely follows official data. UN import/export data are identical to FAO data.
Austria[c]	47	13,759	High	High	Official sales statistics compiled by ISS are used from 1970-2001, after which EC official data are used. UN production and import/export data supports these data for every year available (1970-1995). UN and FAO import/export data are identical.

Netherlands[c]	48	13,451	High	High	Sales data from ISS used from 1970-2001 are nearly identical to EC figures used from 2002-2014 in nine years of overlap. Production figures from UN, ERC, and Euromonitor are all very similar, as are import/export estimates by UN and FAO. However, large export volumes, often exceeding yearly production and imports render these figures extremely volatile.
Azerbaijan	49	13,370	Moderate	Moderate	Official ANS production data are available for every year (1996-2015). ERC, Euromonitor, and CIS production estimates all mirror the official data. Official import data are used for every year available (2001-2015), and UN import export data are used for 1996-2000. ERC data fully support these official figures, except for 1996 and 1997, where import volumes are estimated to be half the UN figures.
Nepal	50	13,075	Moderate	Moderate	UN production data with FAO import/export data are used for 1970-1984 and 200-2004, while gaps in production data from 1985-1999 are filled by data compiled by Karki et al from Ministry of Finance and industry sources. ERC production figures are used from 2005-2013. Although a 2012 NCME sales estimate is somewhat lower than ERC data, there is only a single year of data available. Import/export data from UN are used for every year available (2009-2015) and supports FAO data, with the exception of one anomalously high FAO export estimate for 2011.
Venezuela, Bolivarian Republic of[b]	51	12,900	Moderate	Moderate	USDA production estimates are used for 1970-1990, after which ERC production and import/export estimates are used. FAO and UN import/export data are similar for years of overlap, but UN offers more complete import data, while FAO offers more complete export data. ERC production estimates are identical to USDA estimates in eight years of overlap, but Euromonitor suggests an increasing production volume in contrast to ERC. No official statistics could be found.

Cuba	52	12,694	Moderate	Moderate	Implied consumption compiled by Dr. Suárez Lugo of the Ministry of Health is the primary data source used for every year available (1980,1985,1990-2013). Implied consumption from CubaStat production and import/export data obtained from both online and hardcopy statistical yearbooks are used for every year they could be obtained. Overlap years (1996-2013) between these two sources are very consistent. Official import/export data are consistent for the few years UN data are available. FAO estimates are consistent for later years, but early export estimates (1970-1989), which have since been revised down, are not reliable, thus creating gaps in early data.
Belgium[c]	53	12,550	High	High	ISS sales data compiled from official sources is used from 1970-2001, and EC sales data from 2002-2014. Sales data are identical for all ten years of overlap. UN production data with FAO import/export data generally support these official figures, although estimates for the late 1980s and early 1990s would suggest higher consumption. Note that trade data should be scrutinized due to grouping with Dutch data in earlier years.
Colombia[b]	54	12,109	Medium	Medium	Official production data from the Encuesta Anual Manufacturera is used from 1992-2014. From 1972-1991, DANE production data are used with UN import/export data. USDA production data, which closely tracks DANE data in 18 years of overlap is used from 1970-1971, and all figures are supported by ERC production data. Import/export data are missing or may be unreliable for some years. FAO import/export are identical to UN figures, except for 1999, where there is an anomalously high UN import figure, and official DIAN import/export figures are nearly identical, except for an export data gap from 2002-2004. The source of relatively high UN/FAO figures for these years is unclear, so these years are excluded from analysis. Finally, DIAN/UN/FAO import figures for 2009-2011 are significantly lower than would be

					implied by other countries' reported exports to Colombia. These years are included for analysis, but should be treated with caution.
Switzerland	55	11,930	High	High	Official sales data from the EZV are used for 1995-2015, and ISS sales data obtained from official sources for every year prior. Fifteen years of overlap are highly consistent, and are supported by UN and ERC production data, with UN and FAO import/export data.
Hungary[c]	56	11,858	High	High	Official EC sales data are used from 2002-2014, and ISS sales estimates compiled from local, USDA, and KPMG sources are used for every year prior. Eleven years of overlap between ISS and EC are consistent and are strongly supported by UN and ERC production data with UN and FAO import/export data.
Bulgaria[b]	57	11,735	Moderate	Moderate	ISS sales data obtained from a WHO source, which is identical to UN production data with FAO and UN import/export data, are used from 1970-2000. UN production data are used in 2001, and EC sales data from 2002-2014. EC sales data are very similar to UN data, with the exception of 2013, where UN figures are higher than other estimates. Euromonitor estimate higher consumption for the few years available, but ERC strongly support the use of EC figures. Note that data are extremely unstable due to imprecise measurement.
Moldova, Republic of[a]	58	11,565	Moderate	Low	Production figures from CIS, UN, and two Moldovan Statistical Agency series (one production index) are all largely consistent. UN import/export data, which is identical to FAO for every year of overlap, is used for every year except 1997. 1996 is omitted because export volumes from both sources exceed import and production volume for that year. ERC data support later figures but estimate lower levels of consumption from 1997-2001.
Malaysia[b]	59	11,315	High	Moderate	UN production and import/export data are used from 1970-1989, after which production data derived from official Malaysia Statistics production index are used. Every

					year of overlap with UN production data (1995-2013) is almost identical to official data. Euromonitor import/export data are identical to UN data, and FAO have very similar estimates. ERC sales estimates follow a different trend, but many data points appear to be imputed. There is a severe drop in consumption in 1999 observed in UN, Malaysia Statistics, Euromonitor, and FAO data, so this year is dropped.
Lebanon	60	10,974	Moderate	Low	Official CAS sales data are available from 2000-2011 and 2014-2015. Official CAS production data are used for 2012-2013, and UN production data for 1997-1999 and 1970-1973. UN import/export data is used for each of these years, and are nearly identical to FAO and ERC data. ERC consumption estimates tack these figures closely, except for 1997-1998, so earlier data are not used in the absence of confirmatory data.
Bosnia and Herzegovina	61	9,625	High	High	Official BIS production data are used from 1998-2014 is supported by ERC production data. UN import/export data used from 2003-2014 are strongly supported by FAO data, and ERC data are used from 1998-2002. ERC consumption estimates are used from 1991-1997.
Denmark[c]	65	7,702	High	High	Sales data from ISS used from 1970-2001 is nearly identical to EC figures used from 2002-2014 in nine years of overlap. UN production data with UN and FAO import/export data strongly support these official series.
Slovakia[c]	66	7,480	Moderate	Moderate	ISS sales estimates are used from 1993-1996, and ISS legal sales estimates compiled from KPMG sources are used from 1997-2014. UN import/export data are used in 2015, and follow ISS estimates closely beginning in 2008. Volatile consumption estimates prior to this are partially due to domestic production ceasing in 2007. FAO import/export data are nearly identical to UN data, and ERC sales estimates support ISS data.
Croatia[c]	69	6,739	High	High	Sales data compiled by ISS compiled from official CBS sources are available from 1993-2013 and EC from 2002-

					2014. There are minor, but not insignificant differences between these two sources, but UN production and import/export data support the use of ISS. These UN production and import/export data are used for 1991 and 1992. FAO and UN import/export data are identical.
Sweden[c]	72	6,240	High	High	Official SCB sales data compiled by ISS are used from 1970-2001, and EC sales data are used from 2002-2014. These two sources track closely in five years of overlap, except for 2006, where UN import/export data support the use of EC figures. UN and ERC production, and UN and FAO import/export data are all closely aligned.
Tanzania, United Republic of	75	5,000	High	High	Official TNSB data are used for 2004-2012, and UN production data are used for every other year. FAO import/export data are available from 1970-1996, after which UN import/export data are used. ERC consumption estimates strongly support these figures for every year available (1990-2012)
Myanmar	80	4,560	Moderate	Moderate	Production data is of good quality, with official CSO data closely following UN data, but import/export data are of lower quality. Official data are used for every year available (1990,1995,1999-2014). ERC estimates supported these figures closely, so these are used for gaps in data from 1991-1994 and 1996-1998. Alternate import/export data from FAO and UN are sparse and inconsistent, so no data are presented prior to 1990.
Ireland[c]	86	4,128	High	High	Sales data compiled by ISS from the Central Statistical Office and Revenue Commissioners match European Commission exactly for every overlapping year (2002-2011). Sales data are supported by generally consistent production data, although outliers in imports (2003) and exports (1992) lead to two years of inconsistent data.
Czechoslovakia	N/A	N/A	Moderate	Moderate	Czechoslovakia is analyzed as a unit from 1970-1992. ISS sales data are developed from official sources and USDA estimates. UN production figures closely mirror these estimates. Factoring in FAO import/export estimates

					would raise consumption estimates, but the quality of these imputed figures is questionable, so ISS estimates are used.
USSR	N/A	N/A	High	High	The USSR is analyzed as one entity from 1970-1995. ISS sales data compiled from USDA figures is supported by local sources, and totals are consistent with UN production data combined with USDA import/export data.
Yugoslavia	N/A	N/A	High	High	Yugoslavia is analyzed as one entity from 1970-1990. ISS data, compiled from USDA and local sources, are strongly supported by UN production data.
