# Supplementary material for: Cigarette consumption estimates for 71 countries from 1970 to 2015: systematic collection of comparable data to facilitate quasi-experimental evaluations of national and global tobacco control interventions
Source: BMJ. 2019 Jun 19;365:l2231. doi: 10.1136/bmj.l2231 (PMC6582269; doi:10.1136/bmj.l2231)
Supplement: Supplementary file 4 — Web appendix D: Data collected and verified across countries and years [file hofs048711.ww4.pdf]

Appendix D. Data collected and verified across countries and years

Country	Country Information		Number of years of data collected			
	FCTC Date	Population 2014	Production	Imports	Exports	Consumption
Algeria	Ratified: 30 Jun 2006	27,924,456	24	35	26	16
Argentina	Signed: 25 Sep 2003	32,094,143	45	40	37	37
Armenia	Acceded: 29 Nov 2004	2,441,458	18	18	18	17
Australia	Ratified: 27 Oct 2004	19,179,830	44	44	45	45
Austria	Ratified: 15 Sep 2005	7,295,673	45	39	45	45
Azerbaijan	Acceded: 1 Nov 2005	7,498,994	18	20	20	17
Bangladesh	Ratified: 14 Jun 2004	111,451,025	25	45	38	22
Belarus	Ratified: 8 Sep 2005	7,992,598	18	18	18	17
Belgium	Ratified: 1 Nov 2005	9,326,839	45	34	45	45
Bosnia and Herzegovina	Acceded: 10 Jul 2009	3,291,340	23	23	18	18
Brazil	Ratified: 3 Nov 2005	157,628,688	25	46	28	46
Bulgaria	Ratified: 7 Nov 2005	6,191,839	45	44	45	45
Canada	Ratified: 26 Nov 2004	29,860,626	35	39	44	45
Chile	Ratified: 13 Jun 2005	14,124,866	0	45	41	30
China	Ratified: 11 Oct 2005	1,132,635,009	27	46	45	45
Colombia	Acceded: 10 Apr 2008	35,972,292	24	45	46	42
Croatia	Ratified: 14 Jul 2008	3,616,756	24	18	23	23
Cuba	Signed: 29 Jun 2004	9,488,691	26	45	38	39
Czech Republic	Ratified: 1 Jun 2012	8,967,629	22	16	22	22
Czechoslovakia	N/A	N/A	23	22	22	8
Denmark	Ratified: 16 Dec 2004	4,680,441	45	38	45	45

Egypt	Ratified: 25 Feb 2005	60,098,630	24	45	45	42
Estonia	Ratified: 27 Jul 2005	1,113,037	19	13	19	19
France	Approved: 19 Oct 2004	52,262,503	45	39	45	45
Germany	Ratified: 16 Dec 2004	70,144,789	46	44	45	45
Greece	Ratified: 27 Jan 2006	9,392,650	45	44	45	45
Hungary	Ratified: 7 Apr 2004	8,445,822	45	39	45	43
India	Ratified: 5 Feb 2004	917,035,016	24	45	44	46
Indonesia	Not a participant	183,355,151	26	43	45	44
Iran, Islamic Republic of	Ratified: 6 Nov 2005	59,712,480	25	44	46	46
Ireland	Ratified: 7 Nov 2005	3,665,975	45	37	45	45
Italy	Ratified: 2 Jul 2008	51,525,967	45	39	45	45
Japan	Accepted: 8 Jun 2004	110,321,293	44	45	45	45
Kazakhstan	Ratified: 22 Jan 2007	12,806,379	18	18	19	19
Korea, Democratic People's Republic of	Ratified: 27 Apr 2005	19,653,913	24	24	28	19
Korea, Republic of	Ratified: 16 May 2005	42,828,380	25	45	45	45
Lebanon	Ratified: 7 Dec 2005	4,219,242	26	26	25	18
Lithuania	Ratified: 16 Dec 2004	2,500,826	20	12	19	19
Malaysia	Ratified: 16 Sep 2005	22,407,752	24	46	46	45
Mexico	Ratified: 28 May 2004	90,175,817	24	46	45	46
Moldova, Republic of	Ratified: 3 Feb 2009	3,424,954	18	19	19	19
Morocco	Signed: 16 Apr 2004	24,633,887	44	36	46	41
Myanmar	Ratified: 21 Apr 2004	38,503,196	24	45	28	27
Nepal	Ratified: 7 Nov 2006	18,737,059	1	44	39	17

Netherlands	Accepted: 27 Jan 2005	14,051,206	45	43	45	45
Nigeria	Ratified: 20 Oct 2005	99,522,327	24	21	42	9
Pakistan	Ratified: 3 Nov 2004	119,931,318	24	27	39	45
Philippines	Ratified: 6 Jun 2005	67,175,64	25	44	45	45
Poland	Ratified: 15 Sep 2006	32,833,383	45	45	35	25
Portugal	Approved: 8 Nov 2005	8,917,102	45	39	45	45
Romania	Ratified: 27 Jan 2006	16,603,346	45	29	26	25
Russian Federation	Acceded: 3 June 2008	119,908,242	19	19	19	19
Saudi Arabia	Ratified: 9 May 2005	21,929,58	24	0	46	42
Serbia and Montenegro	Ratified: 8 Feb 2006	7,425,951	3	25	24	24
Slovakia	Ratified: 4 May 2004	4,601,167	22	14	23	23
Slovenia	Ratified: 15 Mar 2005	1,762,776	24	14	23	23
South Africa	Ratified: 19 Apr 2005	37,976,334	46	44	45	45
Spain	Ratified: 11 Jan 2005	39,366,146	45	43	45	45
Sweden	Ratified: 7 Jul 2005	8,037,959	45	35	45	45
Switzerland	Signed: 25 Jun 2004	6,988,340	46	39	45	45
Syrian Arab Republic	Ratified: 22 Nov 2004	11,975,289	25	42	37	21
Taiwan	N/A	20,156,45	45	24	30	30
Tanzania, United Republic of	Ratified: 30 Apr 2007	28,456,00	23	44	45	45
Thailand	Ratified: 8 Nov 2004	55,516,634	24	45	45	37
Tunisia	Ratified: 7 Jun 2010	8,531,430	24	45	39	38
Turkey	Ratified: 31 Dec 2004	57,352,620	44	44	31	30
USSR (Former)	N/A	N/A	26	26	26	26
Ukraine	Ratified: 6 Jun 2006	38,367,853	18	19	19	19

United Kingdom	Ratified: 16 Dec 2004	52,894,193	46	44	45	45
United States	Signed: 10 May 2004	258,370,118	46	46	45	45
Uzbekistan	Acceded: 15 May 2012	21,030,084	18	18	18	18
Venezuela, Bolivarian Republic of	Ratified: 27 Jun 2006	21,970,389	24	44	44	37
Viet Nam	Ratified: 17 Dec 2004	70,984,334	24	40	24	21
Yugoslavia	N/A	N/A	21	21	0	0
