# Supplementary material for: Cigarette consumption estimates for 71 countries from 1970 to 2015: systematic collection of comparable data to facilitate quasi-experimental evaluations of national and global tobacco control interventions
Source: BMJ. 2019 Jun 19;365:l2231. doi: 10.1136/bmj.l2231 (PMC6582269; doi:10.1136/bmj.l2231)
Supplement: Supplementary file 5 — Web appendix E: Glossary of all acronyms used in the database [file hofs048711.ww5.pdf]

Appendix E. Glossary of all acronyms used in the database

ABS: Australian Bureau of Statistics
ADB: Asian Development Bank
ADIC: Alcohol and Drug Information Centre (Ukraine)
AMAR: Statistical Centre of Iran
ANS: Azerbaijan National Statistics
ASEAN: Association of Southeast Asian Nations
BBS: Bangladesh Bureau of Statistics
BIR: Bureau of Internal Revenue (Philippines)
BIS: Bosnia Institute for Statistics
CAPMAS: Central Agency for Public Mobilization and Statistics (Egypt)
CAS: Central Administration of Statistics (Lebanon)
CBSSYR: Central Bureau of Statistics (Syria)
CDS: Central Department of Statistics (Saudi Arabia)
CEFP: Centro de Estudios de las Finanzas Públicas (Mexico)
CIS: Commonwealth of Independent States
CSA: Central Statistical Agency (Ethiopia)
CSB: Central Statistical Bureau (Latvia)
CSO: Central Statistical Office (Poland)
CSO: Central Statistics Organization (Afghanistan)
CSO: Central Statistical Organisation (India)
CSO: Central Statistical Organization (Myanmar)
DANE: Departamento Administrativo Nacional de Estadística
DGI: Dirección General Impositiva (Uruguay)
DIAN: Dirección de Impuestos y Aduanas Nacionales de Colombia
DoC: Department of Commerce (India)
DOSM: Department of Statistics Malaysia
EAM: Encuesta Anual Manufacturera (Colombia)
EC: European Commission
ECOWAS: Economic Community of West African States
EIM: Encuesta Industrial Mensual (Mexico)
EMIM: Encuesta Mensual de la Industria Manufacturera (Mexico)
ERC: European Research Council
Euro: Euromonitor
EZV: Eidgenössische Zollverwaltung (Switzerland)
FAO: Food and Agriculture Organization
FTC: Federal Trade Commission (United States)
GSO: General Statistics Office (Vietnam)
HMRC: HM Revenue & Customs (United Kingdom)
HNZ: Health New Zealand
IIP: Index of Industrial Production (India)
INE: Instituto Nacional de Estadística (Bolivia)
INE: Instituto Nacional de Estadística (Chile)
INEGI: Instituto Nacional de Estadística y Geografía (Mexico)
INEI: Instituto Nacional de Estadística e Informática (Peru)
INPES: Institut National de Prévention et d'Éducation pour la Santé (France)
INSSE: National Institute of Statistics (Romania)

INTABACO: Instituto del Tabaco (Dominican Republic)
IPI: Industrial Production Index (Turkey)
ISS: International Smoking Statistics
KazStat: Ministry of National Economy of the Republic of Kazakhstan Committee on Statistics
MAGP: Ministerio de Agricultura Ganadería y Pesca
Min Fin: Ministry of Finance (Indonesia)
Min of Agr: Ministry of Agriculture & Rural Development (Indonesia)
MinFin: Ministry of Finance (Thailand)
MoF: Ministry of Finance (Turkey)
NBS: National Bureau of Statistics (Moldova)
NBS: National Bureau of Statistics (Nigeria)
NBS: National Bureau of Statistics (Tanzania)
NBSC: National Bureau of Statistics of China
NSO: National Statistical Office (Mongolia)
NTA: National Treasury Administration (Taiwan)
ONE: Oficina Nacional de Estadísticas (Cuba)
ONS: Office National des Statistiques (Algeria)
SARS: South African Revenue Service
SAT: Superintendencia de Administración Tributaria (Guatemala)
SBP: State Bank of Pakistan
SIAVI: Sistema de Información Arancelaria Vía Internet (Mexico)
SORS: Statistical Office of the Republic of Serbia
Stat.kg: National Statistical Committee of the Kyrgyz Republic
TAJ: Tax Administration Jamaica
TBOT: Tax Burden on Tobacco (United States)
TTB: Alcohol and Tobacco Tax and Trade Bureau (United States)
TTM: Thailand Tobacco Monopoly
TurkStat: Turkish Statistical Institute
UKRSTAT: State Statistical Service of Ukraine
UN: United Nations
UNSD: United Nations Statistics Division
USDA: United States Department of Agriculture
WHO: World Health Organization
